# Trends in the Prevalence of Diabetes, 2015–2024 - KNHANES

**DOI:** 10.56786/PHWR.2026.19.42.4

**Published:** 2026-11-05

**Authors:** Boram Kim

**Affiliations:** Division of Health and Nutrition Survey and Analysis, Department of Chronic Disease Prevention and Control, Korea Disease Control and Prevention Agency

In 2024, the prevalence of diabetes (among adults aged ≥19 years, age-standardized) was 10.6%. The prevalence was 13.3% among males and 7.8% among females, representing increases of 1.3%p and 0.9%p, respectively (Figure 1). The prevalence of diabetes increased with age. Among males, the age-specific prevalence was highest in those in their 60s (35.5%), followed by those aged ≥70 years (34.8%). Among females, the prevalence was highest in those aged ≥70 years (28.5%) (Figure 2).

## Supplementary Material



## Figures and Tables

**Figure 1:**
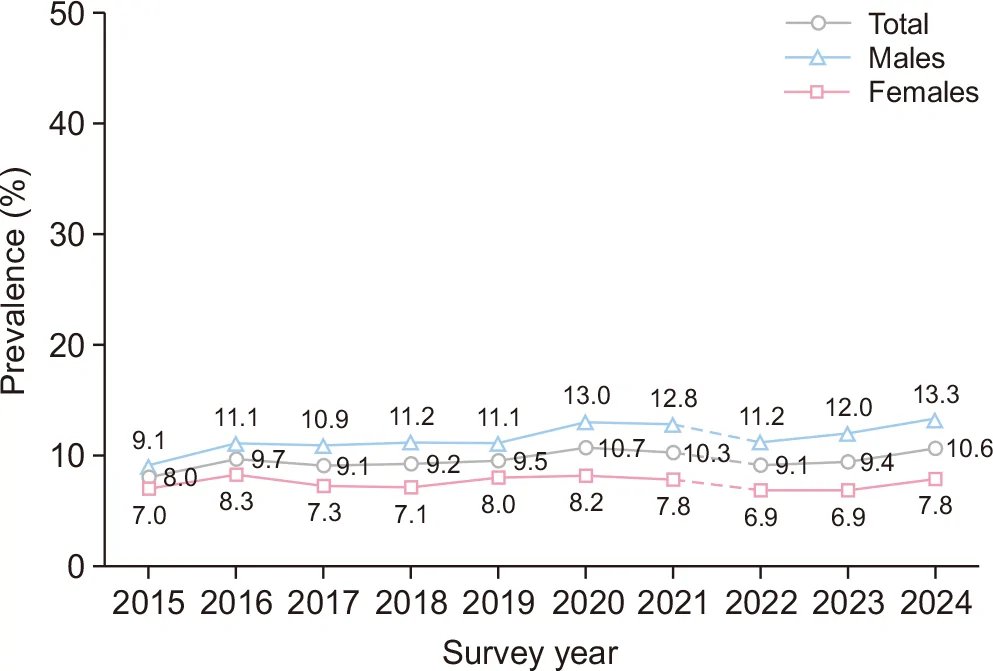
Trends in the prevalence of diabetes, 2015–2024

**Figure 2:**
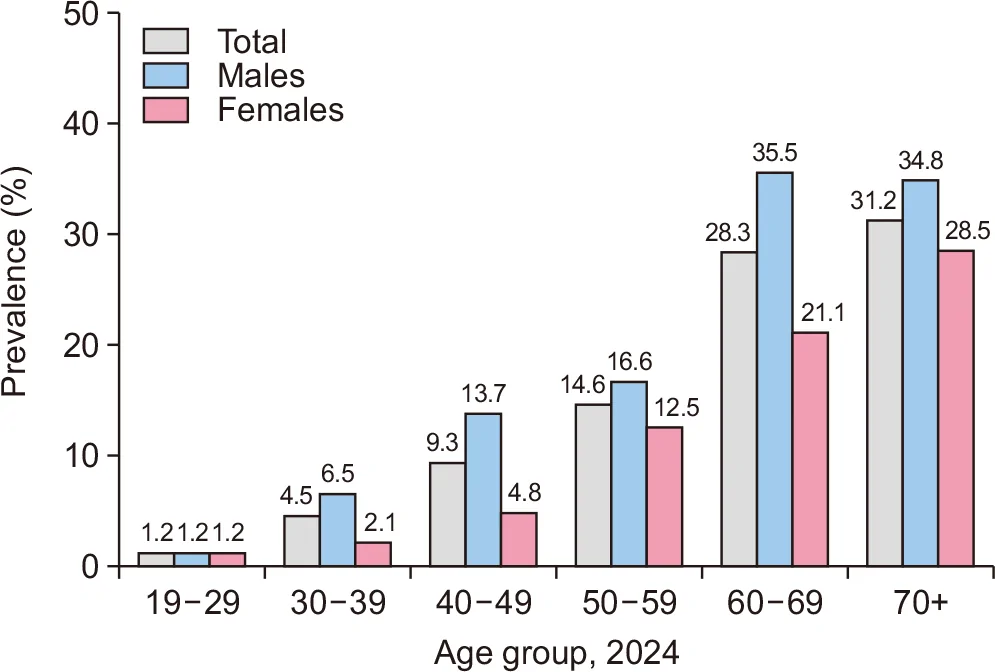
Prevalence of diabetes by age group, 2024

