# Supplementary material for: Trends in the Prevalence of Diabetes, 2015–2024 - KNHANES
Source: Public Health Wkly Rep. 2026 Nov 5;19(42):1787–8. doi: 10.56786/PHWR.2026.19.42.4 (PMC13740381; doi:10.56786/PHWR.2026.19.42.4)
Supplement: Translation

# Translation: 당뇨병 유병률 추이, 2015–2024년 - 국민건강영양조사

**Authors:** 보람 김

**Affiliations:** 질병관리청 만성질환관리국 건강영양조사분석과

2024년 기준, 당뇨병 유병률(19세 이상, 연령표준화)은 10.6%였고, 남자는 13.3%, 여자는 7.8%로 각각 1.3%p, 0.9%p 증가하였다(그림 1). 당뇨병 유병률은 연령이 높을수록 증가하여 남자는 60대, 70대 이상에서 35.5%, 34.8%, 여자는 70대 이상에서 28.5%로 각각 연령별 유병률이 높았다(그림 2).

## Figures and Tables

**그림 1:** 당뇨병 유병률 추이, 2015–2024년

**그림 2:** 연령대별 당뇨병 유병률, 2024년
